# Transition from Coherent to Stochastic electron heating in ultrashort relativistic laser interaction with structured targets

**DOI:** 10.1038/s41598-017-01677-5

**Published:** 2017-05-03

**Authors:** G. Cristoforetti, P. Londrillo, P. K. Singh, F. Baffigi, G. D’Arrigo, Amit D. Lad, R. G. Milazzo, A. Adak, M. Shaikh, D. Sarkar, G. Chatterjee, J. Jha, M. Krishnamurthy, G. R. Kumar, L. A. Gizzi

**Affiliations:** 1 Intense Laser Irradiation Laboratory at INO-CNR, Via G. Moruzzi 1, Pisa, Italy; 2 INAF–Osservatorio astronomico Bologna, Bologna, Italy; 3 Tata Institute of Fundamental Research, Homi Bhabha Road, Colaba, Mumbai, 400005 India; 4 Istituto per la Microelettronica e Microsistemi, CNR, Catania, Italy; 5 INFN, Pisa Section, Largo B. Pontecorvo 3, Pisa, Italy

## Abstract

Relativistic laser interaction with micro- and nano-scale surface structures enhances energy transfer to solid targets and yields matter in extreme conditions. We report on the comparative study of laser-target interaction mechanisms with wire-structures of different size, revealing a transition from a *coherent particle heating* to a *stochastic plasma heating* regime which occurs when migrating from micro-scale to nano-scale wires. Experiments and kinetic simulations show that large gaps between the wires favour the generation of high-energy electrons via laser acceleration into the channels while gaps smaller than the amplitude of electron quivering in the laser field lead to less energetic electrons and multi-keV plasma generation, in agreement with previously published experiments. Plasma filling of nano-sized gaps due to picosecond pedestal typical of ultrashort pulses strongly affects the interaction with this class of targets reducing the laser penetration depth to approximately one hundred nanometers. The two heating regimes appear potentially suitable for laser-driven ion/electron acceleration schemes and warm dense matter investigation respectively.

## Introduction

Relativistic interaction of an ultrashort laser pulse with micro- or nano-structured targets has recently stimulated a large interest for possible application in many fields, including Inertial Confinement Fusion^1^, laser-driven ion/electron acceleration^2, 3^ or warm dense matter creation^4^. The reason for such an interest lies in a more efficient absorption of the laser energy, if compared to a flat target. In the more favourable cases^5^ absorption can be as high as 90% and the enhancement in absorption is mainly due to the extended penetration depth of laser light into the nanostructured target compared to the collisionless skin depth of a flat target, resulting in a much longer interaction time.

Depending on the target geometry, improved laser-plasma coupling can result in volumetric heating of dense matter up to extreme temperatures^4^, stronger X-ray emission^6^ and/or efficient generation and guiding of energetic electrons/ions^7–12^.

A pioneering work in this area is that of Murnane *et al*.^13^ where gold gratings and gold nanoclusters were introduced to enhance target absorptivity. Gordon *et al*.^14^ and Kulcsar *et al*.^15^, then showed a significant enhancement of X-ray emission by using nanostructured surfaces. Since then several target geometries have been tested both in experiments and in numerical simulations, in a wide range of laser intensities from 10^14^ to 10^22^ W/cm^2^, including wires/nano-brush^4, 6, 11, 12^, nano-spheres^2, 16^, gratings^17^, nano-holes^18^, nano/microtubes^3, 7^. However, in spite of the considerable amount of experimental data, a satisfactory knowledge of the key control parameters is still missing, thereby limiting the exploitation of this class of targets for applications. This is also due to the many experimental parameters affecting the Laser Target Interaction regime of published experiments, the most relevant being the morphology of the nanostructures and the laser temporal contrast ratio (i.e. the ratio of the peak intensity to the pedestal intensity), often poorly known.

The critical role played by the geometry of the structures can be easily understood by comparing their characteristic dimensions with the typical scales affecting the interaction process such as the collisionless skin depth, the spatial excursion of quivering electrons in the laser field, the laser wavelength and the density scale-length, usually in the range between 10 nm and 1 μm. It is therefore reasonable to expect that when the dimensions of the structures fall below such values, the interaction will change dramatically. From an experimental viewpoint an ideal investigation path would require a parametric study of the interaction by changing gradually the target geometry. Because of the limitations in making suitable targets with tuneable structure dimensions, this approach is not feasible. Furthermore, in spite of the major recent enhancements of the temporal contrast of laser systems, experiments at high laser intensities are always systematically affected by premature plasma formation that can modify the target prior to the time of peak intensity. In fact, while ns-scale amplified spontaneous emission can be suppressed to levels below plasma formation threshold, the inherent picosecond pedestal will inevitably affect the interaction, giving rise to a preformed plasma with a characteristic scale-length specific of each laser system and partially dependent on target materials. Just as an example, for a relativistic pulse of peak intensity of 10^19^ W/cm^2^, a contrast of 10^6^ just a few ps before the laser peak can produce a pre-plasma with a scale-length of the order of 100 nm, which can be larger than the gap between the structures in the target. Moreover, the plasma formation threshold of nanostructured targets is usually much lower than that of flat targets, because of the higher absorption efficiency^19^.

In this paper we show the effects of nanostructure dimensions on the interaction at relativistic intensities, using silicon wires targets. Nano-sized wires/gaps and micro-sized wires separated by hundreds of nanometers are used to investigate absorption and hot electron generation via dedicated experiments and two dimensional particle-in-cell (2D PIC) simulations. In the experiments, laser target interaction is investigated by characterizing the hot electrons emitted in the backward direction and by systematically comparing the resulting electron spectra with those obtained by using a flat silicon target. Compared with forward escaping fast electrons, backward emitted hot electrons (BWE) are not affected by the interaction with the bulk target substrate and the refluxing inside it, so their properties can be easily referred to the interaction region and modelled by PIC codes. We show that wires gap and size significantly affect laser interaction with the electrons; while nano-wires/gaps, strongly affected by any residual pre-plasma, feature an efficient laser absorption via low-energy, few keV electrons and rapidly generate a hot dense plasma, micro-wires/gaps yield a cooler plasma and a high-energy electron component.

## Experimental setup

The experiment was carried out at the Tata Institute of Fundamental Research TIFR laboratory in Mumbai, using a Ti:Sapphire CPA laser system that delivers 800 nm, 25 fs laser pulses at a maximum energy of 600 mJ (~20 TW).

**Figure 1:**
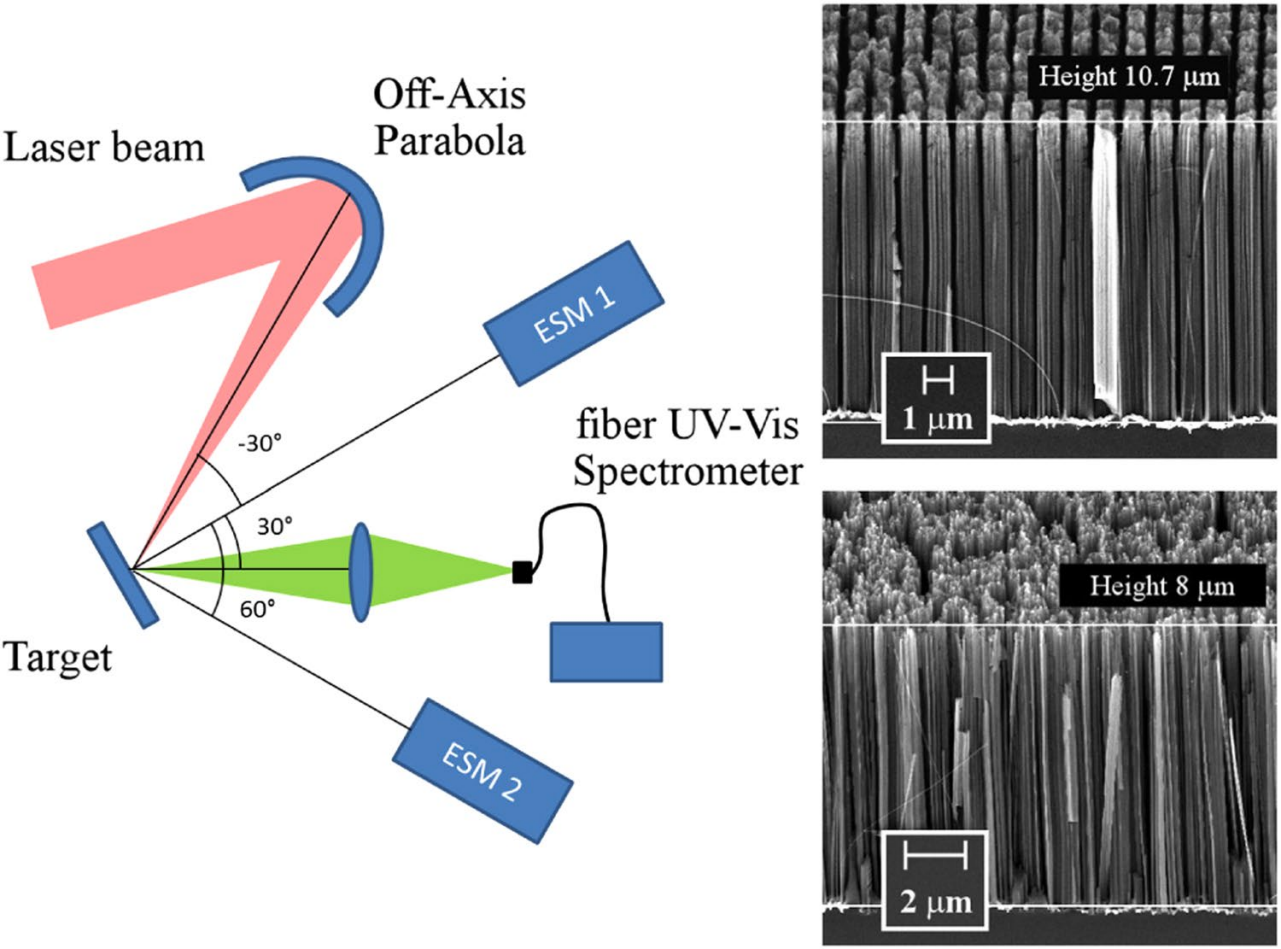
Experimental setup (left). SEM images of MicroWires (MW) (right top) and of NanoWires (NW) (right bottom) targets.

The p-polarized laser beam was focussed on the target with an incidence angle of 30° to an elliptical spot of ~10 × 15 *μ*m FWHM by means of an *f*/2.5 off-axis parabolic mirror (Fig. 1). The temporal profile of the laser pulse was measured by a third order cross-correlation technique and showed a contrast value of the main peak with respect to the ns-long amplified spontaneous emission (ASE) of ~3 × 10^9^ and with respect to a 3 ps pedestal of ~10^6^. The laser irradiance on the target at the best focus was varied in the range $I{\lambda }^{2}=(1.5\,\mbox{--}\,3)\cdot {10}^{18}\,W\,\mu {m}^{2}\,c{m}^{-2}$ $({a}_{0}=1\mbox{--}1.4)$.

Spectra of hot electrons emitted at angles of 0° and 60° were measured by two absolutely calibrated electron spectrometers (ESMs) as sketched in Fig. 1. The ESMs consisted of a 0.1 T dipole magnet and an image plate and detected electrons in the energy range (0.1–7.0) MeV.

Three types of silicon targets were used including optically polished FS (Flat Silicon), NanoWires (NW) and MicroWire (MW) targets. Scanning Electron Micrographs (SEM) of the targets are shown in Fig. 1. The MW targets consisted of pillars of length ~10 *μ*m and 800 nm diameter regularly distributed on a Si surface in a grid and separated by 200 nm gaps (fill factor = 0.64). The lateral walls of the pillars were partially ragged by the fabrication process. The NW targets were formed by nanowires of length ~8 *μ*m and diameter ∼10–20 nm, randomly distributed onto a Si surface, separated by 10–50 nm, resulting in a fill factor in the range 0.04–0.2. The MW and NW targets used in our study, therefore, differ both in the size of the wires and of the gaps.

Light scattered in the specular direction was collected and analysed by a compact spectrometer (350–800 nm) via an optical fiber to monitor the occurrence of three-halves harmonic (*λ* ~ 533 nm), which is here used a signature of preplasma formation^19^.

## Results

### Preplasma and target structures

The formation of a preplasma before the arrival of the main laser peak can damage the structures on the target surface and fill the gaps between them, strongly affecting the interaction of the main peak. A meaningful understanding and modelling of the experimental observations therefore requires a careful characterization of such a preplasma, experimentally or by means of numerical simulations. A mean to accomplish this task is to monitor the formation of three-halves laser harmonic in the emission spectra.

The (3/2)ω_0_ emission arises from the mixing of laser light with electron plasma waves produced by two-plasmon decay (TPD) instability^20^. TPD occurs in the underdense plasma at an electron density *n*_*c*_ */*4, *n*_*c*_ being the critical density, and needs a sufficiently large density scalelength ${L}_{pp}={n}_{e}/(d{n}_{e}/dx)$ to grow, usually ${L}_{pp} > {\lambda }_{0}$ for ultrashort laser irradiation^21, 22^. Therefore, the detection of 3/2*ω*_0_ emission can be used as an indirect indicator of the presence of a pre-plasma^19^. At the lowest laser irradiance explored here ($I{\lambda }^{2}=1.5\cdot {10}^{18}\,W\,\mu {m}^{2}\,c{m}^{-2}$), the 3/2ω_0_ harmonic was never detected in our experiment, suggesting than ${{L}}_{{pp}}\ll {\lambda }_{0}$ during the main peak interaction, as expected, considering the large ASE contrast. A short pre-plasma in the nm range is however expected to be generated by the ps pedestal that overcomes the plasma formation threshold. The order of magnitude of *L*_*pp*_ can be estimated by assuming that the pre-plasma formation occurs 3 ps before the peak of the pulse, where the contrast ratio is approximately 10^6^, and successively expands at the ion-acoustic wave speed. Considering a pre-plasma temperature ≈10 *eV* and *Z* = 3, as given by hydrodynamic simulations, we obtain *L*_*pp*_ ≈ 35 *nm*. This value is in the range of the separation of the NWs (10–50 nm) suggesting that tiny structures with gaps of tens of nanometers are likely to be strongly affected by such a small, ubiquitous pre-plasma. In contrast, the laser interaction with MW targets is expected to be marginally perturbed by this effect.

### Energy spectra of hot electrons

**Figure 2:**
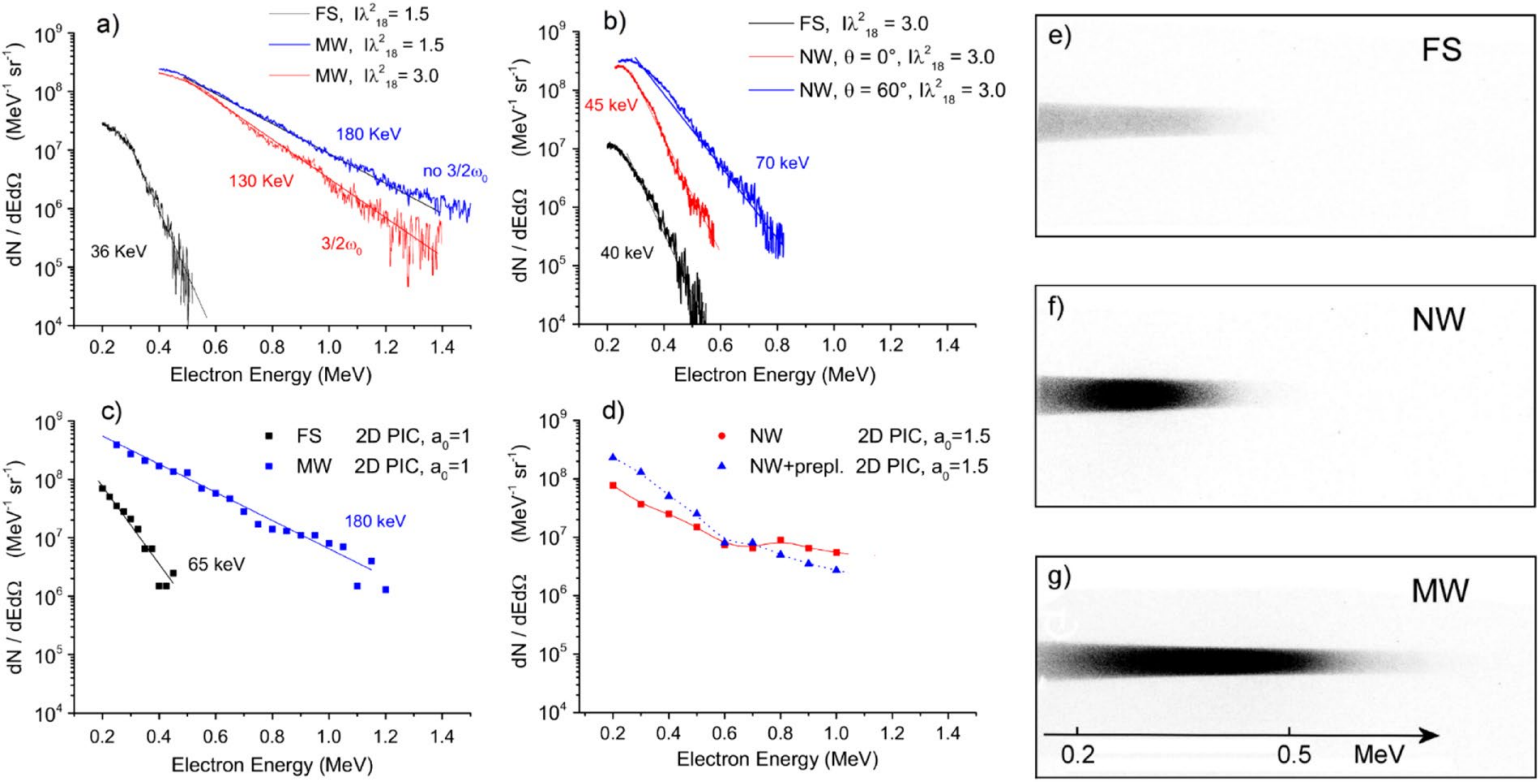
Experimental (**a**,**b**) and simulated (**c**,**d**) spectra of BW hot electrons and raw traces on the imageplate for the different targets (**e**,**f**,**g**). (**a**) FS and MW targets (θ = 0°), $I{\lambda }^{2}=1.5\cdot {10}^{18}\,W\,\mu {m}^{2}\,c{m}^{-2}$ and $I{\lambda }^{2}=3\cdot {10}^{18}\,W\,\mu {m}^{2}\,c{m}^{-2}$; (**b**) FS (θ = 0°) and NW (θ = 0° and 60°) targets, $I{\lambda }^{2}=3\cdot {10}^{18}\,W\,\mu {m}^{2}\,c{m}^{-2}$; (**c**) FS and MW targets, 2D PIC at *a*_0_ = 1, t = 100 fs; (**d**) NW targets, 2D PIC at *a*_0_ = 1.5, t = 100 fs. The 2D PIC spectrum obtained by filling the gaps between NWs with a pre-plasma of density 10 n_c_ is also reported; (**e**,**f**,**g**) $I{\lambda }^{2}=3\cdot {10}^{18}\,W\,\mu {m}^{2}\,c{m}^{-2}$; θ = 0°. For a fruitful comparison of the simulated with the experimental spectra the number of electrons given by 2D simulations has been rescaled by a factor given by the extension of the 2D box in the Z direction and by the solid angle.

Energy spectra of hot electrons for the MW target irradiated at $I{\lambda }^{2}=1.5\cdot {10}^{18}\,W\,\mu {m}^{2}\,c{m}^{-2}$ detected in the direction normal to the target are shown in Fig. 2a. They show a significantly larger flux with respect to the flat target. Also the temperature *T*_*hot*_, calculated by fitting the spectra with an exponential distribution, is found to be as high as 180 keV for MW targets and 36 keV for flat targets, showing a dramatic five-fold increase. Similar results were obtained from spectra measured at 60° from the target normal.

To check the role of a short pre-plasma on the observed rise of hot electrons, we repeated the measurements at twice the laser irradiance, where a larger *L*_*pp*_ would be expected. At $I{\lambda }^{2}=3\cdot {10}^{18}\,W\,\mu {m}^{2}\,c{m}^{-2}$, a broad and weak 3/2ω_0_ emission centred at ~535 nm was detected for MW targets while it was not detected in FS targets. This result suggests that gaps between MWs could be partially filled by underdense plasma during the ps pedestal. In these circumstances, 3/2ω_0_ emission could be explained by the occurrence of TPD instability along the plasma channels between the wires. Electron spectra measured with MW at $I{\lambda }^{2}=3\cdot {10}^{18}\,W\,\mu {m}^{2}\,c{m}^{-2}$ reveal a less efficient generation of high-energy electrons characterized by a lower *T*_*hot*_ (Fig. 2a). This suggests that the damage of the wire-structure over the surface, associated to the partial filling of the gaps, results in a less efficient laser-plasma coupling and electron acceleration.

Measurements carried out with NW targets did not show any 3/2ω_0_ emission at any laser irradiance in the range explored. BWE spectra exhibit an amount of hot electrons larger than that generated with a FS target, as shown in Fig. 2b for $I=3\cdot {10}^{18}\,W\,\mu {m}^{2}\,c{m}^{-2}$. However, the enhancement of both flux and temperature of hot electrons is smaller than the values observed for MW targets, particularly for electrons measured in the normal direction. At θ = 0°, the amount of hot electrons is approximately 30 times higher than for a FS target, while *T*_*hot*_ is approximately the same in the measurement uncertainty. The ESM at θ = 60° yielded a larger total flux and a higher temperature of 70 keV, revealing anisotropic generation of hot electrons (Fig. 2b).

### Kinetic simulations and interaction mechanisms

#### MicroWire Targets

Fully kinetic PIC numerical simulations were carried out using the Aladyn^23^ code in 2D Cartesian geometry. The simulated MW target was designed using realistic sizes, l_y_ = 800 nm for solid material and dl_y_ = 200 nm for the gap size. The main pulse impinges on the tip of nanostructures at t = 48 fs. Calculated spectra of high-energy BW electrons for MW and FS targets are reported in Fig. 2c for *a*_0_ = 1. Spectra account for BW electrons (*p*_*x*_ < 0) in front of the target, (i.e. integrated over all the emission angles) at time t = 100 fs, i.e. 52 fs after the main peak impinges on the tip of MWs.

Simulated spectra show a fair quantitative agreement with experimental spectra measured in the corresponding conditions (Fig. 2a, $I{\lambda }^{2}=1.5\cdot {10}^{18}\,W\,\mu {m}^{2}\,c{m}^{-2}$) for the MW target, while *T*_*hot*_ for FS target is to some extent overestimated.

**Figure 3:**
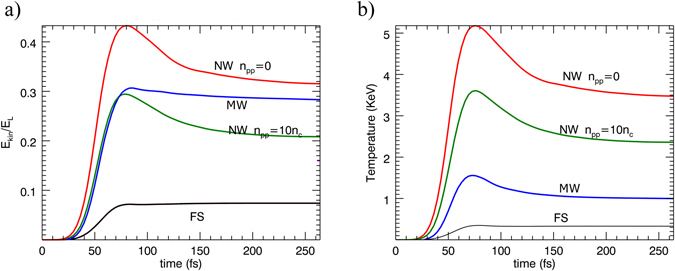
(**a**) Energy absorbed by electrons and (**b**) electron heating, obtained by 2D PIC simulations. The temperature here is calculated as the kinetic temperature averaged over all the electrons into the interaction region, corresponding to a box having a transverse size equal to two times the laser waist and a depth of a 400 nm into the wires.

The simulation results provide us with an effective description of the interaction mechanism. The laser pulse penetrates several microns beyond the tip of the MWs, which results in a significant enhancement of laser absorption (*α* ≈ 0.3) with respect to a planar surface (*α* ≈ 0.07) (Fig. 3a). Since the skin depth is much smaller than the wire diameter, the structures are progressively eroded by the laser pulse. At the end of the interaction (t ~ 100 fs), however, MWs are still well visible, with the gap channels filled by a 2–10 *n*_*c*_ plasma and a skin depth layer of 30–50 nm, at the MW lateral wall, at a plasma density ≈60 *n*_*c*_. At this time, the core of MWs has an electron density of 100–150 *n*_*c*_ and a temperature of around 0.8 keV, likely resulting from cold electron return currents or from electrons accelerated in the gap channels and penetrating the wire. These results suggest that the core is in a warm dense matter regime at the end of the interaction.

According to our simulations, electrons retain almost completely the absorbed energy where only 10% of it is transferred to ions at 200 fs after the laser peak. The large absorption results in a plasma temperature *T*_*e*_ ≈ 1.6 *keV*, significantly higher than the value obtained for FS targets *T*_*e*_ ≈ 300–400 *eV*).

**Figure 4:**
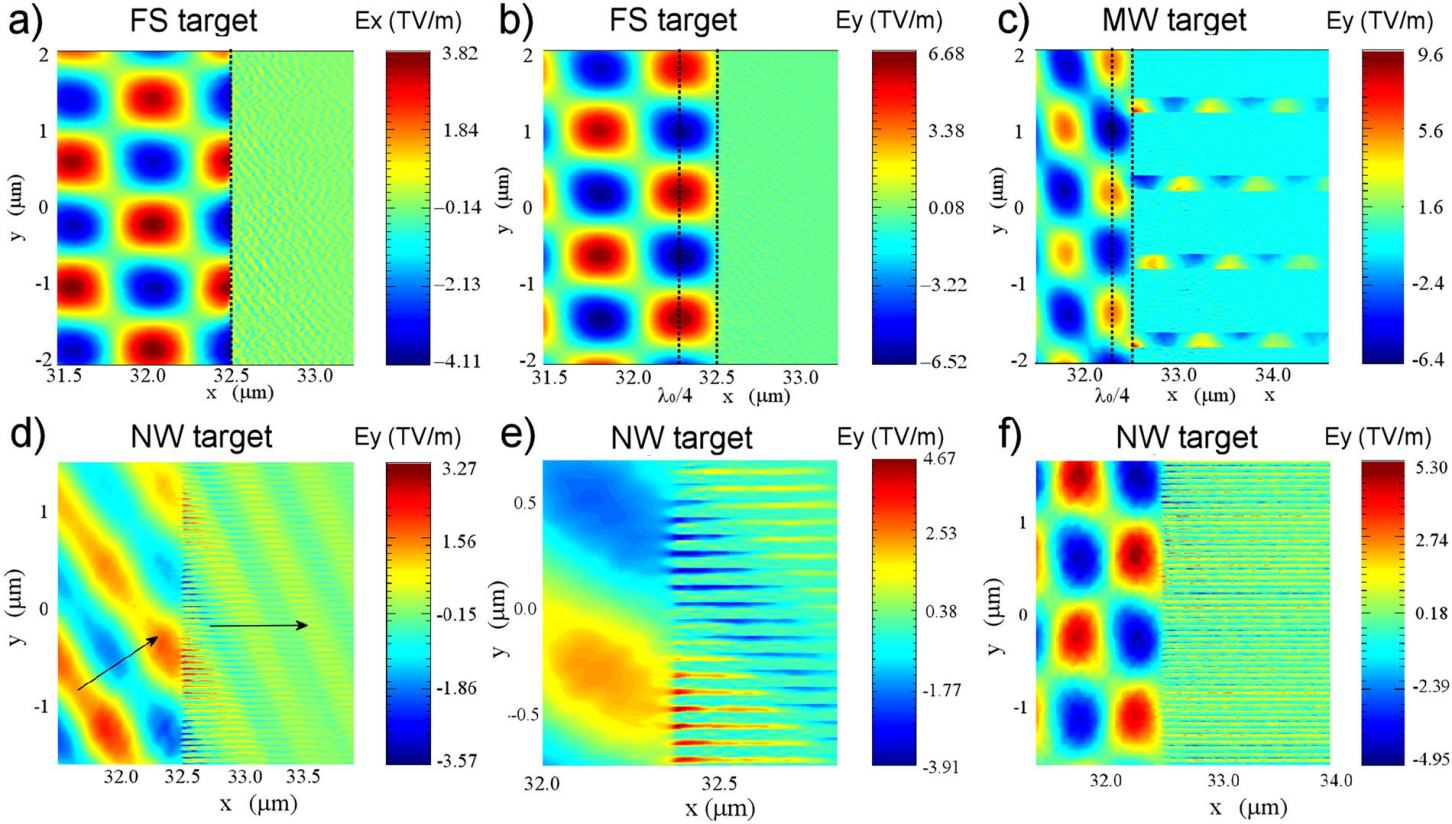
EM field structure for FS (**a**,**b**), MW (**c**) and NW (**d**) (with no preplasma) targets at the time when peak intensity reaches the target surface (t = 48 fs). In (**e**) field enhancement for NW target. (**f**) EM field structure for NW target in the trailing part of the laser pulse (t = 66 fs).

To gain insight into hot electron generation, it is helpful to observe the spatial distribution of the electromagnetic field in FS and MW targets (Fig. 4). For a FS target, *p*-polarization results in a combination of stationary and travelling components. An electric field *E*_*x*_ (~3.4 TV/m at t = 50 fs) near the surface, travelling in the *y* direction, extracts electrons from the target, according to Brunel’s model^24^ (Fig. 4a). The higher electric field ~2*E*_*0y*_ (~6 TV/m) is however localized at a distance ≈*λ*_0 _cos(30°)/4 ≈ 175 *nm* from the surface, produced by the interference of incident and reflecting light and resulting in a standing-wave (SW) pattern along the *x* direction (Fig. 4b). The maximum energy that an electron reaches in vacuum is therefore ~${E}_{{\rm{\max }}}\approx (\sqrt{1+{(2{a}_{0})}^{2}}-1)\cdot {m}_{e}{c}^{2}\approx 590\,keV$, which is much higher than the electron mean oscillation energy in the laser field^25^. This estimation, similar to the one used by May *et al*.^26^ and Kemp *et al*.^27^ for the interaction at normal incidence on a steep overdense target, is in a qualitative agreement with maximum electron energies obtained in our simulated and experimental spectra.

**Figure 5:**
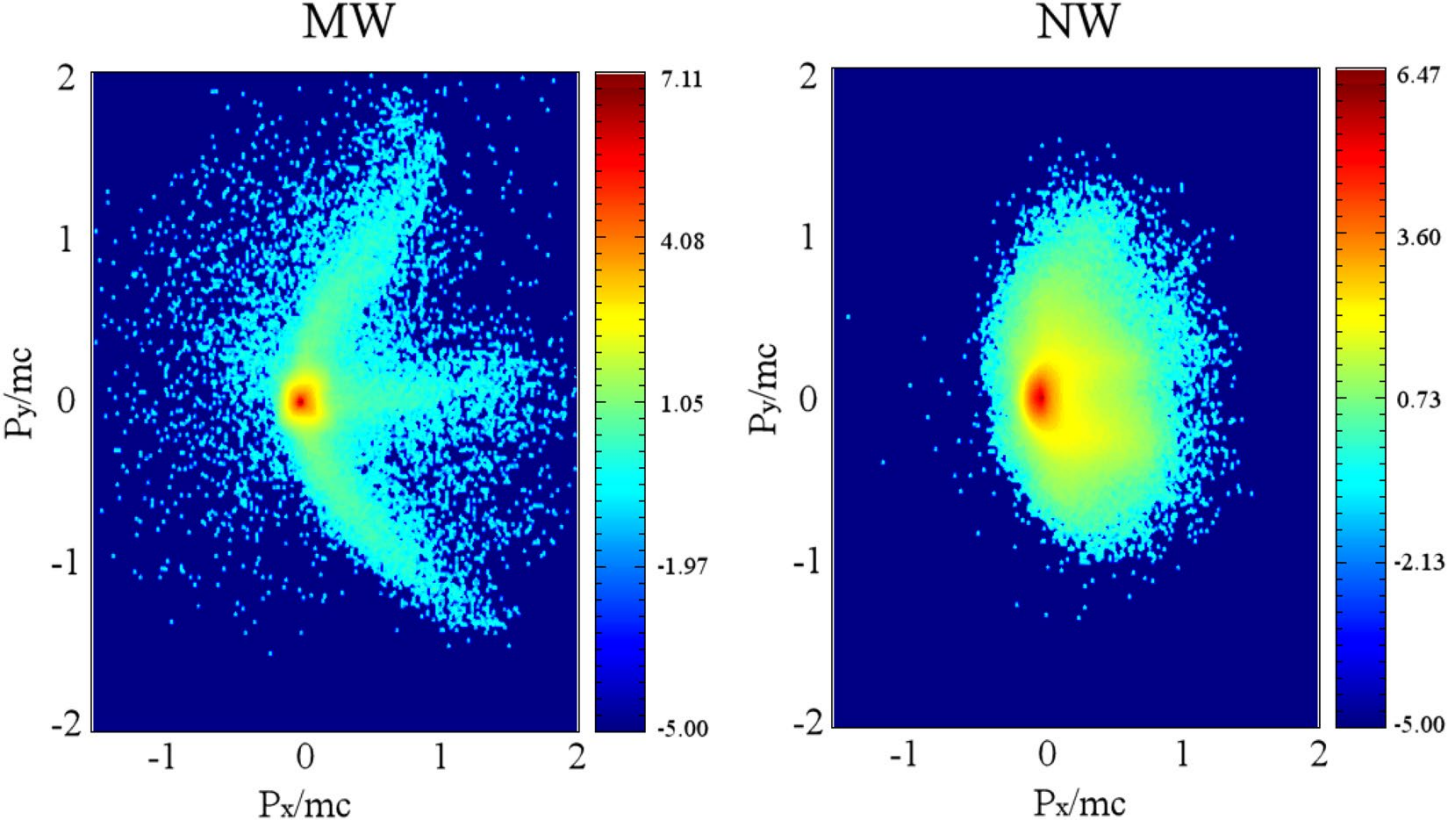
Distribution in logarithmic scale of electron momenta Px vs. Py in the leading part of the laser pulse (t = 40 fs).

For a MW target, the standing-wave pattern in the *x* direction is still present in front of the target; however, because of the larger absorption of the surface, the reflected wave is weaker and therefore the *E*_*y*_ maximum field is slightly lower (~5 TV/m) than in a FS target (Fig. 4c). A travelling EM field (non SW) propagates into the channels and is responsible for high-energy electron generation. The transverse *E*_*y*_ field extracts the electrons from the wire, which successively quiver across the density gradient according to relativistic *JxB* force. Since the amplitude of oscillations is smaller than the channel size, many electrons quiver coherently without reaching the neighbouring wire and acquire a net kinetic energy re-entering into the wire or entering an overdense region. A close look to the *p*_*x*_ vs *p*_*y*_ phase space at the beginning of the interaction (Fig. 5a) shows evidence of a coherent forward acceleration along the channels (central peak) and at angles corresponding to *JxB* quivering. A similar mechanism of forward acceleration of electrons along micro-sized channels was previously discussed by Jiang *et al*.^11^, although at a much higher laser intensity ~10^21^ *W cm* ^−2^. Although most of the hot electrons are accelerated forward, Fig. 5a shows the presence of some backward hot electrons, of similar energy, whose trajectories are probably bent and randomized by magnetic fields. The expected temperature of hot electrons accelerated in underdense plasma is that expressed by the Wilks ponderomotive scaling^28^ ${T}_{hot}\approx (\sqrt{1+{{a}_{0}}^{2}}-1)\cdot {m}_{e}{c}^{2}\approx 194\,keV$, which is in a good agreement with both experimental and numerical results.

### NanoWire targets

NW targets were modelled by considering wires of 20 nm separated by 40 nm gaps. In contrast to the experimental targets, wires are here regularly distributed onto the surface. As shown in Fig. 2d for a pulse with amplitude *a*_0_ = 1.5, the number of generated BW HEs is slightly smaller than the experimental value and their energy distribution, integrated over the entire emission angles, scarcely reproduces the experimental one $(I=3\cdot {10}^{18}\,W\,\mu {m}^{2}\,c{m}^{-2})$. PIC spectra, in fact, exhibit a lower amount of electrons with energy *E* ≤ 500 *keV* and a tail of higher-energy electrons *E* ≥ 800 *keV*, emitted in the normal direction, which is not observed in the experiment. The situation does not appreciably change by varying the fill factor of the structures. The discrepancy with experimental data can be reduced by considering the presence of a tiny preplasma, which rapidly fills the nm-scale gaps and reduces the laser penetration into the target. The effect of preplasma was tested by numerical simulations by partially filling the gaps with a pre-plasma density of *n*_*pp*_ *=* 10 *n*_*c*_ and *n*_*pp*_ *=* 20 *n*_*c*_. In Fig. 2d the BWE spectrum obtained for a preplasma *n*_*pp*_ *=* 10 *n*_*c*_ is reported. The growth of pre-plasma density produces a progressive reduction of the high-energy tail of BW electrons emitted normally to the target, and in the corresponding growth of electrons with energy *E* ≤ 500 *keV*, resulting in a better agreement with the experimental spectrum (blue curve in Fig. 2d). In particular, the temperature of hot electrons with *E* ≤ 500 *keV* decreases from 170 keV in the case where the preplasma is not present to 110 keV and 90 keV for *n*_*pp*_ *=* *10 n*_*c*_ and *n*_*pp*_ *=* *20 n*_*c*_, respectively, approaching the value obtained for a flat target. The flux of hot electrons obtained with a preplasma is in agreement with the experimental results and remains at levels considerably higher than those obtained for a flat target.

A picture of laser target interaction can again be drawn by observing the EM field distribution of the 2D simulations. In case of no initial pre-plasma into the channels, the laser initially enters between the wires (Fig. 4d). A strong spatial modulation like a fine toothed-comb, with an evident field enhancement, is present at the target surface (Fig. 4e), resulting in a much more efficient laser absorption than in a FS target. The large absorption at this time hinders the formation of a clear SW pattern along *x* direction in front of the target.

PIC simulations show however that in the trailing part of the pulse the gaps are partially filled by the plasma, expanding preferentially between the wires rather than in front of the target. At this time, no coherent EM field is observed between the wires (Fig. 4f). The larger laser reflection now results in a clear SW pattern in front of the target, even if the maximum EM field is lower than for a FS target.

In the actual interaction, a tenuous pre-plasma is already present between the wires at the arrival of the main peak making the initial evolution of the fields similar to that observed above in the trailing part of the pulse. The SW-pattern accompanied by a substantial absence of pre-plasma in front of the target, results in the generation of hot electrons with an energy distribution similar to that obtained for a FS, as observed in the normal direction. Here, however, their flux is enhanced by the larger absorption (Fig. 3b) resulting in a larger amount of electrons reaching the SW accelerating fields. The oscillating EM field across the comb teeth, attenuated by the pre-plasma presence, could result in the generation of hot electrons propagating mainly along the surface or at larger angles, as experimentally observed, even if an accurate investigation of hot electron trajectory, accounting for magnetic fields, is here needed. Laser interaction with the wires can be inferred by looking at the phase plot of electron momenta into the interaction layer (Fig. 5b). Similar to the MW, the electrons are extracted by the wires and accelerated by the laser field, but given the small gap between wires, the energy gain of the electrons is limited by the collision with neighbouring wires and their trajectories are randomized. In this way, electrons are stochastically heated to a higher bulk electron temperature and the high-energy tail of forward hot electrons has a much lower temperature than that obtained with a MW target. The target depth where the stochastic heating occurs, however, decreases with the density of the preplasma present into the gaps approaching the interaction conditions obtained by using a flat homogeneous target. For *n*_*pp*_ *=* *10 n*_*c*_ the laser penetration depth reduces approximately to one hundred nanometers resulting also in a reduction of the total laser absorption, from *α* ≈ 0.5 (*n*_*pp*_ *=* *0*) to *α* ≈ 0.29. In spite of the fact that the pre-plasma filling the gaps reduces the laser absorption to levels similar or lower than for MW, the energy is now shared by a lower number of bulk electrons, due to the lower fill factor of the target and to the lower temperature of HE. Therefore, NW targets results in higher plasma temperature, going from *T*_*e*_ ≈ 5.2 *keV* for *n*_*pp*_ = *0* to *T*_*e*_ ≈ 3.6 *keV* for *n*_*pp*_ = 10 *n*_*c*_, calculated in an interaction layer of thickness 400 nm and 100 nm, respectively. These values are in agreement with multi-keV temperatures obtained by Purvis *et al*.^4^, who irradiated Au and Ni nanowires with a relativistic high-contrast laser at $I=5\cdot {10}^{18}\,W\,c{m}^{-2}$. Simulations also show that the abrupt leak of electrons from the nanowire, due to the quivering in the laser EM field, drives its Coulomb explosion, resulting in a considerable and rapid transfer of energy to ions. This energy constitutes 35–40% of the total energy absorbed at 200 fs after the peak of the pulse.

The different interaction regime envisaged for MW and NW targets suggests that a suitable tailoring of the surface can enhance its performance, depending on the desired aim. Additional preliminary results obtained by a parametric investigation with 2D PIC simulations indicate that laser absorption rises for smaller NW sizes and for larger gaps, while keeping the other parameter fixed. Larger values of laser absorption are therefore obtained for smaller fill factors, reaching values *α* > 0.9 for a few-tens of nanometers tiny wires separated by hundreds of nanometers gaps. In this case, only a small fraction of the laser energy is reflected by the tip of the NWs while most of it penetrates between the wires and is absorbed by multiple reflection scattering. Moreover, since the skin depth is only a few tens nanometers, the increase of NW size beyond this value does not provide an increment of electrons capable of absorbing the laser light. These preliminary indications, however, do not account for the possible presence of a tiny preplasma among the wires nor for the effects of 3D geometry on the laser interaction, and therefore need an accurate validation which is out of the scope of the present work.

## Conclusions and Outlook

In conclusion, our comparative study shows for the first time the role of key geometrical parameters in relativistic laser interaction with nanostructured targets. Size and separation of wires were found to be governing parameters in setting the interaction regime while taking into account realistic laser contrast. Gaps between structures play a key role in determining the energy distribution of hot electrons. Larger gaps lead to high-energy hot electrons, generated by coherent laser acceleration into the channels while small gaps give rise to a stochastic heating of the electrons inhibiting the formation of high-energy hot electrons in favour of the formation of a hot, dense plasma. In the first case, the enhanced generation of hot electrons makes targets with large gaps potentially suitable for ion acceleration. In contrast, smaller gaps result in hot plasmas with temperatures reaching several keV, making these targets suitable for warm dense matter investigation and strong shock generation. These targets are however very sensitive to even tiny amount of pre-plasma generated by high-contrast laser pulses, which contribute to prevent efficient acceleration of electrons but also tend to reduce the volumetric character and the efficiency of laser absorption.

## Methods

Particle In Cell simulations were carried out using the Aladyn^20^ code in 2D Cartesian geometry. The intensity of a *p*-polarized laser pulse was modelized by a Gaussian profile in the transverse coordinate with focal spot of 10 μm FWHM. The time profile on the focal plane was given by $I(t)={I}_{0}{\cos }^{4}\,(\pi t/2\tau )$ where 2τ = 70 fs corresponds to a pulse length τ_FWHM_ = 25 fs. The laser pulse enters in the computational (X, Y) box from the left edge, with incidence angle α = 30° with respect to the target normal. The sizes of the numerical box were set to L_x_ = 40 μm and L_y_ = 50 μm and the grid cell to dx = dy = 10 nm, assuring reasonable space-time resolution. The initial density of Si^4+^ ions was set to ${n}_{Si}=4.3\cdot {10}^{22}\,c{m}^{-3}$, corresponding to a plasma electron density ${n}_{e}=100\,{n}_{c}=1.74\cdot {10}^{23}\,c{m}^{-3}$. In the PIC code, 256 (macro) electrons per cell were used. During dynamical laser-plasma interaction, field ionization using the ADK model has been activated. At the intensities here considered, Si ions increases ionization levels from the initial *Z* = 4 to a final *Z* = 8–10.
